# Association between single nucleotide polymorphisms rs12722489 and multiple sclerosis in Iranian patients with multiple sclerosis

**Published:** 2020-01-05

**Authors:** Habib Ahmadi, Vahid Reza Yassaee, Reza Mirfakhraie, Feyzollah Hashemi-Gorji

**Affiliations:** 1 Department of Medical Genetics, School of Medicine, Shahid Beheshti University of Medical Sciences, Tehran, Iran; 2 Genomic Research Center, Shahid Beheshti University of Medical Sciences, Tehran, Iran

**Keywords:** Multiple Sclerosis, Interleukin 2 Receptor Subunit Alpha Protein, Single Nucleotide Polymorphism

## Abstract

**Background:** Multiple sclerosis (MS) is a complex incurable neurodegenerative disease featuring demyelination of neurons, resulting in impairment of neuron impulses. Recently, an association of two single nucleotide polymorphisms (SNP) (rs2104286 and rs12722489) in interleukin 2 receptor subunit alpha (IL2RA) gene was found to be a risk factor of MS in white European population. Therefore, we performed a study to investigate the contribution of these two intronic variations in Iranian patients with MS.

**Methods: **We determined the genotypes of rs2104286 and rs12722489 in patients with MS (n = 100) and in the control group (n = 111). The SNPs were genotyped using tetra-primer amplification refractory mutation system-polymerase chain reaction (T-ARMS-PCR) for both of SNPs. Statistical analysis was performed by SPSS software. Also, odds ratios (ORs) and 95% confidence interval (CI) were calculated.

**Results: **Logistic regression revealed that various genotypes of rs12722489, regarding sex-adjusted effect, yielded meaningful association with MS risk in Iranian patients (OR = 2.67, 95% CI: 1.03-6.90). However, no association was obtained for rs2104286 and rs12722489 with MS.

**Conclusion:** The results confirmed partially the reports in white European population performed recently. However, further investigation in larger scale is necessary to validate our study.

## Introduction

Multiple sclerosis (MS) is a chronic neurodegenerative disease characterized with neuronal demyelination and leads to impaired neuronal impulses consequently affecting the patients with limbs numbness, fatigue, visional complications, etc.^1^

Epidemiological studies have shown that the disease has included approximately 2 million people throughout the world, and according to Iranian MS Association, only 40000 patients with MS have been registered in this country.^2^ Etiologically, MS is categorized in autoimmune diseases, involving both environmental and genetic risk factors in proneness to it.^3^^-^^5^

From the genetics points of view, human leukocyte antigen-DR beta 1 (HLA-DRB1) gene which is located in the chromosome 6p21, for decades was the merit locus identified in giving the risk to MS.^6^ However, with the development of chromatin immunoprecipitation (ChIP)-based techniques, several other important genetic variations, which were unidentifiable previously, have been nominated for the disease. Noteworthy, several genomic polymorphisms including genetic variation in genes such as cytotoxic T-lymphocyte-associated protein 4 (CTLA4), tumor necrosis factor (TNF), and cytochrome P450 family 27 subfamily B member 1 (CYP27B1) have been recognized so far.^7^^-^^10^ Furthermore, the recent genome-wide association studies (GWASs) in European white population harvested other genetic risk factors in susceptibility to MS including interleukin 7 receptor subunit alpha (IL7RA) and interleukin 2 receptor alpha (IL2RA) (also called CD25) genes.^11^

IL2RA gene is located in the chromosome 10p15. It functions as the IL2 specific receptor, assembling the whole IL2R, and plays salient roles such as activation of T lymphocytes. Hence, it has a central situation in the cellular immune system in human body.^12^ This is the basis of the fact that IL2RA gene can be spontaneously a candidate for several immune diseases.^13^

In this line, IL2RA gene polymorphisms, exonic and intronic, were primarily identified to give susceptibility to autoimmune diseases including type 1 diabetes mellitus (DM) in several studies.^14^ IL2RA gene intronic single nucleotide polymorphism (SNP), rs2104286, represented an association with the risk of type 1 DM in previous studies and was founded to correlate with the extent of secretory form of IL2RA protein in subjects.^15^ Its minor allele (G) was recognized to hold protective role from type 1 DM in the white population.

Therefore, this study was placed to produce an association study among Iranian patients with MS to examine whether the two IL2RA intronic variations (rs2104286 and rs12722489) that are potential risk factors for autoimmune diseases are actually associated with MS risk in Iranian population.

## Materials and Methods

This was a pilot case-control study which was approved by the Ethics Committee of Shahid Beheshti University, Tehran, Iran. A total number of 211 Iranian subjects, including 100 patients with MS referred to Sina Hospital, Tehran and 111 healthy people from blood donors in Tehran Blood Transfusion Center as controls, were enrolled (from 2013 to 2014). MS diagnosis was performed according to McDonald criteria. All participants in the investigation primarily gave the informed consents. Data regarding the age of onset of disease at diagnosis and clinical course of the patients were obtained [data regarding patients’ Expanded Disability Status Scale (EDSS) record was not accessible]. Also, in the control group, the individuals who had any background of autoimmune diseases in their families including thyroiditis, diabetes, psoriasis, etc., were excluded of our study. 

Peripheral blood samples of all the participants were obtained, of which, deoxyribonucleic acid (DNA) contents were extracted using salting out method.^16^ To determine the genotype of samples, we used tetra-primer amplification refractory mutation system-polymerase chain reaction (T-ARMS-PCR). Thus, primers for rs2104286 and rs12722489 were designed by Lasergene software (version 7.1, DNASTAR, Madison, WI, USA). The sequence of primers for rs2104286 is as follows: Outer Forward ATCTATCCAATATCTCTCATGCCCCTTT; Inner Forward AAAATTCCTACACAAGCAAACAAACAGC; Inner Reverse GCATAGATATAGTCATGGTAACACAAGGCA; Outer Reverse CCCATGCTCAGTAGATCTTACCACAGAC. As for rs12722489 primers, it is: Outer Forward CAGGGTCTTTGCTAACTCAGTCTAAGCC; Inner Forward TTTCTAGCTATTGGTGACTTATCCAATGA; Inner Reverse CCTGCTCCCTCCAAGACCACTCATAC; Outer Reverse TTCTCCTCCTCACCAGGTTATAAGTTGC. T-ARMS-PCR was done using Gene Fanavaran Company (Tehran, Iran) Taq DNA Pol 2x Master Mix Red (Ampliqon Denmark) in a total volume of 25 μl. This process was accomplished for the polymorphisms under conditions, consisting of primary denaturation for 3 minutes at 95 °C, followed by 35 cycles of 95 °C for 20 seconds, annealing temperature (62.5 °C for rs2104286, 60 °C for rs12722489) for 45 seconds, following a 5-minute extension time at 72 °C in Labcycler 48 Gradient (SensoQuest Gottingen, Germany). To proceed, PCR products were detected on the 3% agarose with red green stain for 1 hour in Tris/Borate/EDTA (TBE) 1X buffer in voltage of 90 V in electrophoresis instrument (Lab FAQS Roche, Germany). Furthermore, to confirm the genotyping task, 10 percent of the subjects’ samples, randomly, were sequenced in Applied Biosystems (ABI) sequencer 3130 equipment. 

All statistical analysis was done in SPSS software (version 20, IBM Corporation, Armonk, NY, USA) and Excel 2010 software while performing Hardy-Weinberg equilibrium by online Hardy-Weinberg calculator software. The association of different alleles of rs2104286 with MS was examined by SNP STATS Analyzer online program, as done for rs12722489. Fisher’s exact test also was applied for evaluating the relation of status of patients’ clinical course at the age of onset with the different genotypes of alleles. Finally, logistic regression was used, since it was to evaluate the actual effect of sex-adjusted status in subjects on interaction between the categorical variables, regarding different distribution of genotypes of the SNPs studied.

## Results

In the present study, both the SNPs examined obeyed Hardy-Weinberg equilibrium (no P-value below 0.05). Demographic data, corresponding the subjects studied (Table 1) showed that the means of the variables of height and weight between case and control groups were not statistically significant with P-value greater than 0.05, but regarding the age, it was significant (P = 0.04). Also, age of onset at diagnosis was 27.5 ± 8.0 years in participating patients.

Data regarding clinical course of the patients showed that the highest percent of patients with MS were put into the relapsing remitting MS (RRMS) (87%) category and the remaining fell into the other categories [Secondary Progressive MS (SPMS) = 10% and Primary Progressive MS (PPMS) = 3%]. The results are shown in table 1.

**Figure 1:**
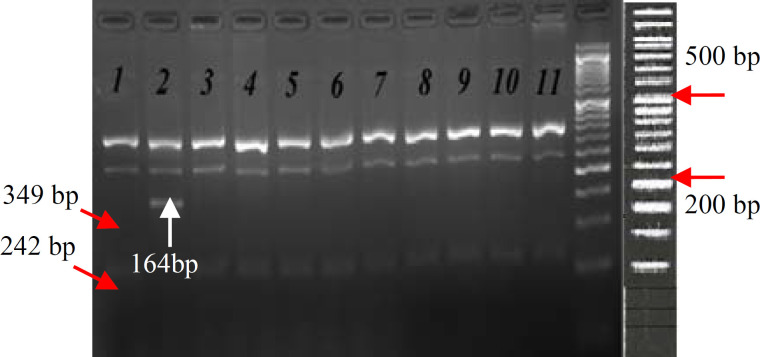
Agarose gel electrophoresis of tetra-primer amplification refractory mutation system-polymerase chain reaction (T-ARMS-PCR) products, corresponding to different genotypes of rs2104286 in the subjects. The lane 1 shows an individual of A/A genotype, the lane 2 denotes A/G genotype.

**Figure 2:**
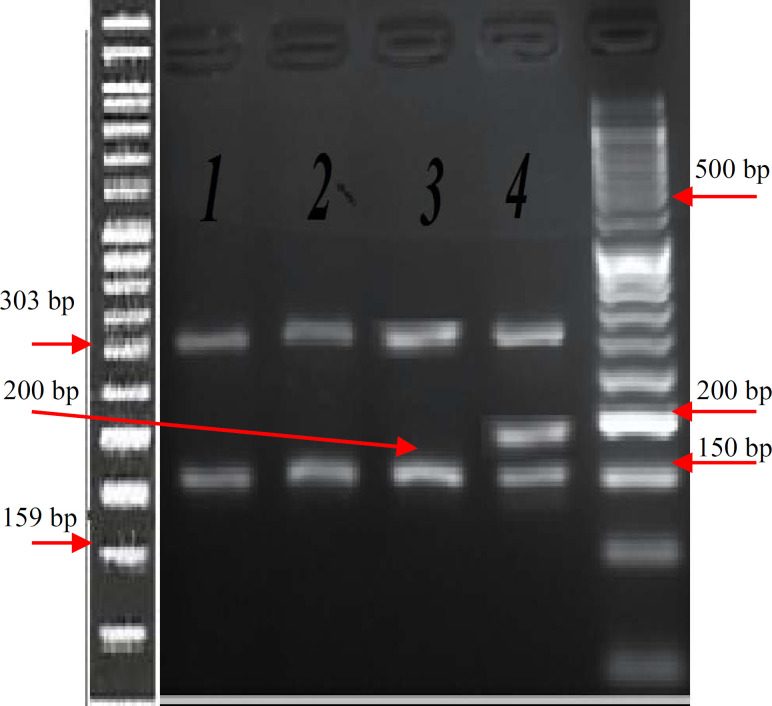
Agarose gel electrophoresis of tetra-primer amplification refractory mutation system-polymerase chain reaction (T-ARMS-PCR) products, corresponding to rs2104286 in the subjects. The lane 1 shows an individual of G/G genotype; the lane 4 shows an individual of A/G genotype.

As for the genotyping task, the allele typing of sample was performed which was revealed in figures 1 and 2. 

The genotype distribution and allele frequencies of the SNPs in patients with MS and the control group are presented in table 2. Pertaining data revealed that for rs2104286, genotype A/A had the highest frequency (n = 144) and the genotype G/G was in the lowest frequency (n = 2) in our subjects (Table 2). Also, similar calculus for rs12722489 showed the highest frequency for genotype G/G (n = 190), while not observing genotype A/A among the subjects (Table 2). Observing distribution of rs2104286 alleles in subjects revealed neither relation to MS risk nor different genotypes of rs12722489, as shown in table 2 for supposed modes of inheritance (P > 0.05). Moreover, the confirmation of the genotyping performed in the current study for both rs2104286 and rs12722489 was a validation to the results and was carried out by Sanger sequencing. 

**Table 1:** Demographic characteristics of the patients and control groups according to sex

**Variable**		**Case (n = 100)**	**Control (n = 111)**	**P**
Age (year) (mean ± SD)		33.12 ± 16.61	54.51 ± 9.84	0.04
Age of onset (year) (mean ± SD)		27.51 ± 8.38	ND	ND
Sex [n (%)]	Male	41 (41.0)	41 (36.9)	NS
	Female	59 (59.0)	70 (63.1)	NS
Weight (kg) (mean ± SD)	Male	72.37 ± 16.35	80.16 ± 18.27	NS
	Female	67.41 ± 9.79	74.32 ± 10.38	NS
Height (cm) (mean ± SD)	Male	171.91 ± 14.24	172.63 ± 6.97	NS
	Female	166.40 ± 9.52	165.73 ± 7.45	NS

SD: Standard deviation; ND: Not determined; NS: Not specified

**Table 2:** Frequencies of interleukin 2 receptor subunit alpha (IL2RA) genotypes and alleles in patients with multiple sclerosis (MS) and controls

**Model**	**Genotype**	**Patients with MS (n = 100)**	**Controls (n = 111)**	**OR (95% CI)**
		**n (%)**	**n (%)**	
rs2104286				
Codominant	AA	70 (70.0)	74 (66.7)	1.00
	AG	29 (29.0)	36 (32.4)	1.17 (0.65-2.11)
	GG	1 (1.0)	1 (0.9)	0.95 (0.06-15.42)
Dominant	AA	70 (70.0)	74 (66.7)	1.00
	AG-GG	30 (30.0)	37 (33.3)	1.17 (0.65-2.09)
Recessive	AA-AG	99 (99.0)	110 (99.1)	1.00
	GG	1 (1.0)	1 (0.9)	0.90 (0.06-14.58)
Overdominant	AA-GG	71 (71.0)	75 (67.6)	1.00
	AG	29 (29.0)	36 (32.4)	1.18 (0.65-2.11)
Log-additive	-	-	-	1.15 (0.66-1.99)
	A allele	169 (84.5)	184 (82.9)	
	G allele	31 (15.5)	38 (17.1)	
rs12722489				
	GG	85 (85.0)	103 (92.8)	1.00
	AG	15 (15.0)	8 (7.2)	0.44 (0.18-1.09)
	G allele	185 (92.5)	214 (96.4)	
	A allele	15 (7.5)	8 (3.6)	

MS: Multiple sclerosis; OR: Odds ratio; CI: Confidence interval

To analyze any probable interaction of rs2104286 and rs12722489 and any likely role of the patients’ status including sex status in our subjects which might affect the results, we exploited logistic regression test. The results obtained showed that the rs2104286 A/G genotype vs. A/A genotype decreased the chance of the disease by 8%; nevertheless, it was not statistically meaningful. In contrast, there was a significant association for rs12722489 allele distribution, regarding sex-adjusted variable, and rs2104286 different genotypes with MS risk [odds ratio (OR) = 2.67, 95% confidence interval (CI): 1.03-6.90, P = 0.043].

## Discussion

The current study aimed to examine the association of two intronic polymorphisms of IL2RA gene (rs2104286 and rs12722489) with MS. IL2RA gene has a pivotal role in gearing up cellular immune system. Results taken by this investigation revealed moderate effects of the SNPs of IL2RA gene in susceptibility to MS for Iranian people. However, readily remarkable impacts of the IL2RA gene polymorphisms in several studies previously done in western Caucasians highlighted this gene as a responsible one for MS risk. In this study, it was observed that there was no sign of direct association of different alleles of the SNPs with MS. 

The association of the two SNPs of IL2RA first intron, rs2104286 and rs12722489, accomplished recently by Consortium of MS Centers in 2008 while performing an association study on American and British cohorts, gave a hint into susceptibility causes to MS. Ongoing studies in a few cohorts concerning again western white populations in a large number of samples confirmed the results obtained by the consortium for both polymorphisms as well. In addition, an association between rs2104286 and MS was reported in previous studies.

Remarkably, the investigations performed in western Caucasians nominated the SNPs rs2104286 and rs12722489 as the most substantial ones for the populations. In the present study, no association was identified between rs2104286 polymorphism and MS risk. Alsahebfosoul et al. found no relation between the SNP and the MS in Iranian population.^   17 ^ In contradiction to it, a previous functional study proved a relation between its different alleles and soluble secretary form of IL2RA protein.^15^ However, this intronic polymorphism interrupts neither is a regulatory element in IL2RA gene, nor presents in any protein product of the gene. If looked deeply into it, IL2RA paradoxically acts as a receptor for regulatory T (Treg) cells, which function as repressor cells of immune system. On the other hand, according to Hinks et al. rs791589 which is located in the first intron of the putative gene is the most significant variation, thus presenting other substantial variants in the gene. So, it seems that several variations in this region could contribute to different levels of IL2RA protein resulting in susceptibility to MS in patients.^13^ But, lack of relation in our study simply could be due to limited sample size as opposed to GWASs of Europeans. Moreover, selecting the participants randomly in our investigation from different ethnicities of Iranian population, contrary to the European studies, may be the other culprit. 

In this study, an association, not much significant as ones detected in European studies, was identified between different genotypes of rs12722489 and risk of MS. On the other hand, rs12722489 had been considered a haplotype of rs2104286, the actual putative responsible polymorphism in donating proneness to MS in at least western ancestors. Therefore, more investigations could be done to evaluate these results for determining actual haplotype in Iranian population. 

What emphasizes more on the importance of studying the IL2RA gene polymorphisms is the matter of the implication of some new drugs, specifically those targeted for blocking the gene product. Daclizumab, a monoclonal antibody synthesized to target IL2RA on lymphocytes, promised a good treatment approach for patients with MS in the clinical trials recently performed, for instance a current successful one in Iranian MS patients. In this way, it may be of value to imbed studies in the future based on the relation of IL2RA SNPs and outcomes of these types of clinical trials in patients with MS. 

Meanwhile, the heterogeneity of MS makes an obstacle in interpretation of results in studies with small sample size. It is from the literature that there is only a 2.5% likelihood of having been affected by MS in the siblings of the patients with MS.^18^

## Conclusion

One cannot insist on results of genetic association studies on MS risk without sobering. Furthermore, different results which are obtained by different studies could be attributable to the probable various genes of pathogenicity in different populations and different environments. For example, temperature changes or nutritional habits and distress conditions can pose people to autoimmune disease risk. Therefore, it seems that many other studies in whole genome scale are to be performed to recognize all genes responsible for MS pathogenesis.

## Notes:

**How to cite this article:** Ahmadi H, Yassaee VR, Mirfakhraie R, Hashemi-Gorji F. Association between single nucleotide polymorphisms rs12722489 and multiple sclerosis in Iranian patients with multiple sclerosis. Curr J Neurol 2020; 19(1): 26-31.
